# Association between Mineralocorticoid Receptor Antagonist and Mortality in SARS-CoV-2 Patients: A Systematic Review and Meta-Analysis

**DOI:** 10.3390/healthcare10040645

**Published:** 2022-03-30

**Authors:** Jean Kim, Kyle Miyazaki, Parthav Shah, Landon Kozai, Jakrin Kewcharoen

**Affiliations:** 1 Department of Internal Medicine, University of Hawaii, Honolulu, HI 96813, USA; kylesmiy@hawaii.edu (K.M.); parthavs@hawaii.edu (P.S.); lkozai@hawaii.edu (L.K.); 2 Division of Cardiovascular Medicine, Loma Linda University Health, Loma Linda, CA 92354, USA; jakrinkewcharoen@gmail.com

**Keywords:** COVID-19, SARS-CoV-2, coronavirus, mineralocorticoid receptor antagonist, aldosterone antagonist, spironolactone, meta-analysis

## Abstract

Since the onset of the severe acute respiratory syndrome coronavirus 2 (SARS-CoV-2) pandemic, various potential targeted therapies for SARS-CoV-2 infection have been proposed. The protective effects of mineralocorticoid receptor antagonists (MRA) against tissue fibrosis, pulmonary and systemic vasoconstriction, and inflammation have been implicated in potentially attenuating the severity of SARS-CoV-2 infection by inhibiting the deleterious effects of aldosterone. Furthermore, spironolactone, a type of MRA, has been suggested to have a beneficial effect on SARS-CoV-2 outcomes through its dual action as an MRA and antiandrogen, resulting in reduced transmembrane protease receptor serine type 2 (TMPRSS2)-related viral entry to host cells. In this study, we sought to investigate the association between MRA antagonist therapy and mortality in SARS-CoV-2 patients via systematic review and meta-analysis. The systematic review was performed according to the Preferred Reporting Items for Systematic Reviews and Meta-Analyses (PRISMA) guidelines. MEDLINE and EMBASE databases were searched for studies that reported the incidence of mortality in patients on MRA with SARS-CoV-2 infection. Pooled odds ratio (OR) and 95% confidence interval (CI) of the outcome were obtained using the random-effects model. Five studies with a total of 1,388,178 subjects (80,903 subjects receiving MRA therapy) met the inclusion criteria. We included studies with all types of MRA therapy including spironolactone and canrenone and found no association between MRA therapy and mortality in SARS-CoV-2 infection (OR = 0.387, 95% CI: 0.134–1.117, *p* = 0.079).

## 1. Introduction

Severe acute respiratory syndrome coronavirus 2 (SARS-CoV-2) infection was declared a pandemic by the World Health Organization (WHO) in March 2020 and has created a global health crisis with recurrent surges [1,2,3]. More than 350 million SARS-CoV-2 infection cases have been reported worldwide with over 5 million related deaths from the multisystemic disease. The pathophysiology and targeted treatment modality for SARS-CoV-2 remain unclear, however, and studies are underway.

A mineralocorticoid receptor antagonist (MRA) is an important part of guideline-directed medical therapy in heart failure with reduced ejection fraction and also used in chronic hypertension, primary aldosteronism, and ascites from liver cirrhosis [4,5,6]. MRAs inhibit the activity of aldosterone, the final effector of the renin–angiotensin–aldosterone system (RAAS), by blocking aldosterone receptors and preventing their deleterious effects such as electrolyte imbalance, endothelial dysfunction, smooth muscle cell proliferation of the blood vessels, glomerular injury in the kidneys, and myocardial inflammation and fibrosis. Spironolactone is a type of MRA that also has antiandrogenic properties, and studies have implicated its potential effect on decreasing the severity of SARS-CoV-2 infection. In this study, we aimed to investigate the effect of MRA use on mortality in patients with SARS-CoV-2 infection through systematic review and meta-analysis.

## 2. Materials and Methods

### 2.1. Search Strategy

Two investigators (J.K. and K.M.) independently conducted a systematic search for published studies indexed in the MEDLINE and EMBASE databases from inception to December 2021, utilizing a search strategy that included the terms “SARS-CoV-2”, “COVID”, “COVID-19”, “coronavirus”, “mineralocorticoid receptor antagonist”, and “aldosterone antagonist”. Patients with all disease statuses and methods of conditioning regimens were included. There was no restriction on the types of mineralocorticoid receptor antagonist used, patients’ ethnicity, gender, race, age, data sources, or study location. Review articles, case reports, commentaries, and letters were excluded. A manual search for additional pertinent studies using references from the retrieved articles was also performed.

### 2.2. Study Inclusion Criteria

The eligibility criteria for inclusion of studies are the following:

(1) Randomized controlled trials (RCTs), cohort studies (prospective or retrospective), case-control studies, and cross-sectional studies that reported the incidence of mortality in patients who were infected with SARS-CoV-2 who were on MRA compared to those who were not on MRA therapy;
(2) Odds ratio (OR), hazard ratio (HR), or risk ratio (RR), and its corresponding 95% confidence intervals and *p*-values or sufficient raw data for these calculations had to be provided.

### 2.3. Data Extraction

Two investigators (J.K. and K.M.) independently performed a systematic review of studies following the Preferred Reporting Items for Systematic Reviews and Meta-Analyses (PRISMA) guidelines [7]. A standardized data collection was conducted by obtaining the following information from each study: title, name of authors, year of publication, country of origin, the number of participants in the MRA therapy group, and the control (no MRA) group who have SARS-CoV-2 infection. In addition, information about the type of MRA, patients’ mean age, gender, and comorbid conditions was collected. Any conflict on the data extraction was resolved by the investigators’ consensus following discussions.

### 2.4. Quality Assessment of the Included Studies

The Newcastle–Ottawa quality assessment scale (NOS), ranging from 0 to 9, was utilized to evaluate each study in the following domains: recruitment and selection of the participants, similarity and comparability between the groups, and ascertainment of the outcome of interest among cohort studies [8]. The Cochrane Collaboration tool for assessing risk of bias was used to assess the quality of randomized controlled trial by assigning a score (high, low, or unclear) to individual element from five domains (selection, performance, attrition, reporting, and other).

### 2.5. Statistical Analysis

Meta-analysis of the included studies was performed to determine the pooled effect size with a 95% confidence interval (CI). The outcome of interest was the incidence of mortality in the MRA therapy group versus the control (non-MRA) group with SARS-CoV-2 infection. The heterogeneity of effect size estimates across the studies was quantified using the Q-statistic and the corresponding *p*-value or equivalent using the Higgins I-squared (I2) statistic [9]. In our study, meta-analysis was performed using the random-effects model, and the main results were summarized in a forest plot. To test the robustness of the results, a sensitivity analysis was performed by conducting meta-analyses excluding one study at a time [10]. All meta-analyses were performed using STATA 16 software (StataCorp LLC, College Station, TX, USA).

## 3. Results

### 3.1. Study Search Results

**Figure 1:**
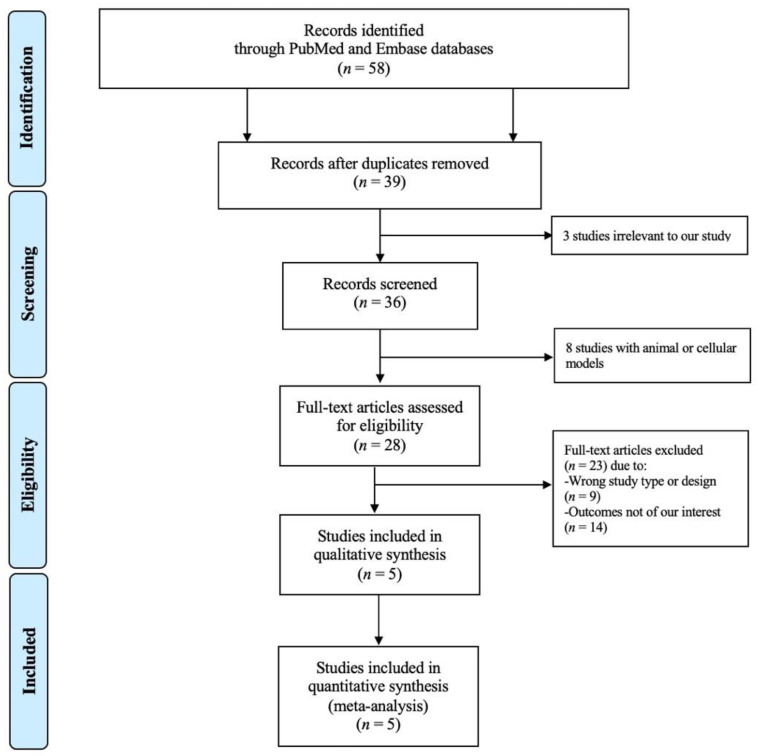
A PRISMA diagram depicting the search methodology and selection process.

Figure 1 shows a PRISMA flow diagram that depicts the process of identification, screening, eligibility, and inclusion or exclusion of the studies. The initial search of the PubMed and EMBASE databases yielded 58 articles. After title and abstract review, a total of 19 duplicate studies were removed, followed by elimination of three studies that were irrelevant to our study and eight studies conducted in animals or cellular models. Subsequently, 28 studies underwent full article review. Of these articles, nine studies were excluded because they were not of the appropriate type or design of study for our analysis, and 14 studies were eliminated as they did not have the outcomes of interest. The final analysis included five unique studies with a total of 1,388,178 subjects.

### 3.2. Description of the Included Studies and Quality Assessment

A total of five studies with 1,388,178 subjects (80,903 subjects received MRA therapy) were included in our meta-analysis (one single-blind randomized-controlled study, two cross-sectional studies, and two case-control studies) [11,12,13,14,15]. The main characteristics of the included studies are described in Table 1. The mean age of subjects of the included studies ranged between about 50 to 70 years old, and the male gender constituted about 50% to 80% of total subjects in the studies. Regarding the type of MRA therapy, two studies utilized spironolactone [12,13], one study used canrenone [14], and two studies did not specify the type of MRA [11,15]. Four studies provided the raw data for the incidence of mortality in patients with SARS-CoV-2 infection who received MRA therapy and those who did not receive MRA therapy and calculations were manually performed to obtain the OR and its corresponding 95% CI. On the other hand, Vicenzi et al. directly provided the value of OR and its corresponding 95% CI [14]. The Newcastle–Ottawa Scale (NOS) of the five studies ranged from 6 to 9 with a mean score of 8, reflecting a high quality of these studies. For the RCT study included in the meta-analysis, the Cochrane Collaboration tool for assessing the risk of bias was used. This showed a low risk of bias in most categories, except for the lack of double-blinding in the study design.

### 3.3. Quantitative Meta-Analysis Results

**Figure 2:**
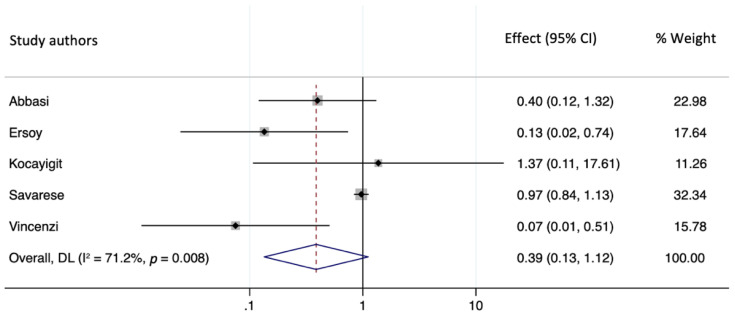
A forest plot showing the OR (odds ratio) for the association of MRA therapy and incidence of mortality in SARS-CoV-2 infection.

We assessed the presence of heterogeneity among the studies in terms of the Q-statistic and the corresponding *p*-value [9]. In the present case of *p* < 0.05, heterogeneity among studies existed. We quantified the degree of heterogeneity by using the I2 statistic which indicated a substantial heterogeneity among the studies (I2 > 50%). We employed the random-effects model to analyze the pooled effect size given substantial heterogeneity [16]. The incidence of mortality in patients with SARS-CoV-2 infection who received MRA therapy was not significantly lower than that of the non-MRA group (OR = 0.387, 95% CI: 0.134–1.117, *p* = 0.079). The forest plot demonstrating the pooled OR comparing mortality in patients with SARS-CoV-2 infection who received MRA therapy and those who did not receive MRA is shown in Figure 2.

### 3.4. Publication Bias

We aimed to investigate potential publication bias via the funnel plot and Egger’s test [17]. However, as we only had five studies included in the analysis, this number was insufficient to reject the assumption of no funnel plot asymmetry; thus, we did not perform a funnel plot or Egger’s test [18,19].

### 3.5. Sensitivity Analysis

To examine the robustness of the pooled OR and 95% CI of our study, sensitivity analyses were undertaken by excluding one individual study at a time [10]. When the study by Savarese et al. was removed, the result was statistically significant. Otherwise, no significant changes were noted when excluding other studies.

## 4. Discussion

The present study is the first systematic review and meta-analysis that investigated the incidence of mortality in SARS-CoV-2 infection who received MRA therapy compared to those who did not receive MRA therapy. Our study included all types of MRA therapy, and the results underscored that MRA therapy does not provide mortality benefit in patients with SARS-CoV-2 infection.

SARS-CoV-2 necessitates transmembrane protease receptor serine type 2 (TMPRSS2) to bind to an angiotensin-converting enzyme 2 (ACE2) receptor via proteolytic processing of the viral spike protein and becomes internalized to the host cell [20,21]. ACE2 has been identified as a key receptor for SARS-CoV-1 and SARS-CoV-2 to enter host cells and is expressed in essential organs including the lungs, heart, brain, and the kidneys in humans [22,23,24]. ACE2 serves as a counterregulatory enzyme of the renin–angiotensin–aldosterone system (RAAS), a cascade of vasoactive peptides that regulates blood pressure and fluid balance in the body, and degrades angiotensin II, thereby inhibiting its adverse effects such as acute lung injury, myocardial remodeling, vasoconstriction, sodium retention, and fibrosis [25,26,27,28]. Upon endocytosis of the viral complex, surface ACE2 levels are downregulated, and can result in angiotensin II accumulation and local activation of RAAS.

The interaction between SARS-CoV-2 and ACE2 has been proposed as a possible factor affecting the viral infectivity. Concerns about RAAS inhibitors affecting ACE2 levels have been previously raised; however, data in humans are limited. Experimental animal models have shown mixed findings on the effects of ACE inhibitors and angiotensin-receptor blockers (ARBs) on ACE2 levels [29,30]. Currently, experts warn about potential adverse clinical outcomes from abrupt withdrawal of RAAS inhibitors, and it is recommended to continue RAAS inhibitors in patients who are otherwise stable and are at risk for or have SARS-CoV-2 infection [31,32]. Recombinant ACE2 has been suggested to possibly normalize angiotensin II levels, and clinical trials are underway to assess whether recombinant ACE2 may restore balance in the RAAS cascade and prevent organ injury in patients with SARS-CoV-2 infection.

Androgen activity has also been implicated in SARS-CoV-2 infectivity and disease severity. Increased levels of circulating androgens contribute to a higher expression of transmembrane proteins such as TMPRSS2 that facilitate viral binding and entry into human cells by modulating viral spike proteins [33,34]. Early reports corroborating these findings have indicated that male gender is an independent risk factor for severe SARS-CoV-2 infection and that children tend to have milder disease courses than adults [35,36,37]. Subsequent studies have also shown that men with androgenic alopecia or anabolic androgenic steroid use, as well as women with hyperandrogenic states such as polycystic ovary syndrome (PCOS), can increase the risk for SARS-CoV-2 infection [38,39,40,41].

Finally, the protective effects of MRAs against tissue fibrosis, inflammation, and vascular dysfunction have been implicated in potentially attenuating the severity of SARS-CoV-2 infection by inhibiting aldosterone [33,42,43]. Previous authors have hypothesized that spironolactone, a type of MRA, may have a beneficial effect on SARS-CoV-2 outcomes through its dual action as an MRA and antiandrogen, resulting in reduced TMPRSS2-related viral entry [38,44,45]. In animal models, spironolactone was also associated with reduction in oxidative stress and lung injury and has been considered a possible therapy in the treatment of COVID-19-related pulmonary fibrosis [42,46,47]. Despite the promising effects of this widely used drug, the effect of spironolactone on morbidity and mortality in patients with COVID-19 remains unclear, as the results of recent studies have been variable [48,49].

In our meta-analysis, we have included studies with all types of MRA therapy including spironolactone and canrenone, and have found that there is no association between MRA therapy and mortality in SARS-CoV-2 infection (OR = 0.387, 95% CI: 0.134–1.117, *p* = 0.079). Despite its known effect of decreasing sympathetic activity, inflammation, tissue fibrosis, and the potential action of hindering viral entry to host cells, MRA therapy did not translate into a significant decrease in mortality in patients with SARS-CoV-2 infection. Mortality was chosen as the outcome of our interest as this particular outcome was consistently reported across the studies. However, it remains to be seen whether MRA therapy has association with other outcomes such as oxygen saturation, need for intubation or vasopressors, duration of hospitalization, and levels of inflammatory markers. Prospective clinical trials are currently underway to further elucidate the relationship between MRA therapy and morbidity and mortality in SARS-CoV-2 infection.

There are several limitations in our study. First, only one RCT was included in the analysis, while other studies were case-control and cross-sectional studies, making our analysis subject to unmeasured confounding factors which are inherent to observational study designs. Moreover, the types of MRA were varied and sometimes not reported in the studies, and the doses of MRA therapy received by patients were not uniform. Recent studies suggest possible beneficial effects of RAAS inhibitors and non-insulin anti-hyperglycemic agents against SARS-CoV-2 infection, and concurrent use of other medications such as angiotensin-converting enzyme inhibitors (ACEi), angiotensin receptor blockers (ARB), dipeptidyl peptidase-4 inhibitors (DPP4i), sodium-glucose cotransporter-2 inhibitors (SGLT2i), and glucagon-like peptide-1 receptor (GLP1) agonists reported in several of the included studies may have confounded the results [50,51,52,53,54]. Subsequently, an elevated heterogeneity was noted in our analysis (I2 = 71.2%, *p* = 0.008), and the random-effects model was employed in our meta-analysis to account for this heterogeneity. It is also notable that, in our sensitivity analysis, the result of the analysis when the study by Savarese et al. was removed was statistically significant. Of note, the study by Savarese et al. involved a much larger number of subjects in a nationwide registry population, compared to that of other studies (Table 1). Ultimately, the sensitivity analysis suggests that the findings of our study may need to be interpreted with caution.

## 5. Conclusions

In conclusion, our meta-analysis does not establish a significant association between MRA therapy and mortality in patients infected with SARS-CoV-2. Based on the lack of definitive benefit of MRA use in SARS-CoV-2 infection, clinicians should be cautious in initiating MRA therapy in these patients. Larger-scale randomized controlled trials with extended follow-up periods are needed to further elucidate the relationship.

## Figures and Tables

**Table 1:** Main characteristics of the included studies (*n* = 5).

Author	Country	Published Year	Study Type	MRA (*n* = 80,903)	No MRA (*n* = 1,307,275)	Mean Age (Years)	Male (%)	HTN (%)	DM (%)	HLD (%)	MRA Type
Abbasi	U.S.	2021	Randomized-controlled trial	50	87	57.0	54.0	31.4	27.7	NR	Spironolactone
Ersoy	Turkey	2021	Case control	30	30	58.8	80.0	NR	NR	NR	Spironolactone
Kocayigit	Turkey	2020	Cross-sectional	5	161	65.8	46.7	100	34.9	16.6	NR
Savarese	Sweden	2020	Cross-sectional	80,788	1,306,958	73.5	52.1	79.8	28.7	NR	NR
Vicenzi	Italy	2020	Case-control	30	39	61.0	72.0	45.0	NR	20.0	Canrenone

Abbreviations: MRA, mineralocorticoid receptor antagonist; HTN, hypertension; DM, diabetes; HLD, hyperlipidemia; NR, not reported.

## Data Availability

The datasets generated and analyzed during the current study are available from the corresponding author on reasonable request.
